# Evaluation of the beneficial effects of pemafibrate in hypertriglyceridemia with or without alcohol drinking (PAR-CHAT: PARmodia-CHikushi Anti-dyslipidemia Trial)^☆^

**DOI:** 10.1016/j.ijcrp.2024.200359

**Published:** 2024-12-13

**Authors:** Yosuke Takamiya, Chiyori Imanaga, Amane Ike, Akira Kawamura, Hidenori Urata

**Affiliations:** Department of Cardiovascular Diseases, Fukuoka University Chikushi Hospital, Japan

**Keywords:** Practicing physician, Liver damage, Low-density lipoprotein cholesterol, Alcohol drinking, Statin treatment

## Abstract

**Purpose:**

To examine the efficacy and safety of pemafibrate in outpatients with hypertriglyceridemia, including alcoholic hypertriglyceridemia.

**Method:**

This multicenter, open-label, prospective observational study (C20-07-009) included outpatients with hypertriglyceridemia being treated with pemafibrate who were registered at Fukuoka University Chikushi Hospital or associated clinics. Endpoints were changes in serum triglyceride (TG) and high-density lipoprotein cholesterol (HDL-C), hepatic biomarkers, and other blood values from baseline to 24 weeks and safety. Patients were compared according to alcohol drinking.

**Result:**

From October 2020 to March 2022, 203 patients were registered at 14 facilities. We analyzed 174 patients (mean age, 65.5 years) with baseline fasting TG values who continued pemafibrate for 24 weeks; 55 % drank alcohol, and 35 % were receiving statins. Median fasting TG was 284 mg/dL (IQR, 228–392 mg/dL) at baseline and decreased significantly to 141 mg/dL (IQR, 108–194 mg/dL) at 24 weeks (p < 0.01), independent of alcohol use (non-drinking group, 259 to 134 mg/dL; daily drinking group, 318 to 169 mg/dL; occasional drinking group, 298 to 158 mg/dL; all p < 0.01). Median HDL-C increased significantly from 46 mg/dL (IQR, 39–53 mg/dL) at baseline to 53 mg/dL (IQR, 45–60 mg/dL) at 24 weeks (p < 0.01). Hepatic biomarkers improved significantly at 24 weeks. Low-density lipoprotein cholesterol (LDL-C) increased significantly overall, but not in patients receiving statins. Side effects throughout the study period included dizziness and nausea (1 patient each).

**Conclusion:**

Pemafibrate improves TG, HDL-C, hepatic biomarkers and hypertriglyceridemia regardless of alcohol consumption and is safe. Increase of LDL-C was suppressed in patients treated with statins.

## Highlights

• We evaluated the efficacy and safety of pemafibrate in hypertriglyceridemia with or without alcohol drinking in a multi-center prospective real world clinical study.
• We evaluated the efficacy and safety of pemafibrate in patients with hypertriglyceridemia with or without alcohol drinking in a multi-center prospective real world clinical study.
• Pemafibrate improved hypertriglyceridemia and liver function regardless of alcohol drinking.
• Pemafibrate combined with statin therapy was safe and suppressed the increased LDL-C due to Pemafibrate treatment.

## 1 Background

In hypertriglyceridemia, a type of dyslipidemia, lipid abnormalities are characterized by an increase in lipoproteins containing a large amount of triglyceride (TG; TG-rich lipoproteins), an increase in small dense low-density lipoprotein (LDL) particles, and a decrease in high-density lipoprotein cholesterol (HDL-C) [[1], [2], [3]]. The disease leads to progression of atherosclerosis and is often associated with metabolic syndrome, hypertension, and type 2 diabetes. A cohort study of approximately 8000 people showed that if hypertriglyceridemia is left untreated, the risk of myocardial infarction, ischemic heart disease, and all-cause mortality increases regardless of sex [4].

Fibrate drugs (e.g., peroxisome proliferator-activated receptor α [PPAR α] agonists), nicotinic acid derivatives, and eicosapentaenoic acid preparations have been used to treat dyslipidemia patients with high TG levels and low HDL-C levels [5]. On the other hand, hydroxymethylglutaryl coenzyme A reductase inhibitors (Statins) therapy is recommended for primary and secondary prevention to stay within LDL-cholesterol management goal of reducing MACE risk [5]. Correcting TG and HDL-C is also considered important because of the remaining high risk of atherosclerosis due to insufficient control of other lipids other than LDL-C [6]. Under these circumstances, fibrate drugs, which lower TG and increase HDL-C, could be useful for correcting residual risks. However, there is a need for new drugs with fewer restrictions on use and an excellent benefit-risk balance, and efforts are being made to develop a drug for dyslipidemias that strengthens the effects of PPARα agonists, as represented by fibrate drugs, but has fewer unfavorable adverse effects.

After binding to the nuclear receptor PPARα, pemafibrate induces a ligand-specific conformational change of PPARα and the expression of a group of genes involved mainly in hepatic lipid metabolism. Pemafibrate was developed in Japan and marketed in June 2018. It is a selective peroxisome proliferator-activated receptor-α modulator (SPPARMα) that improves lipid metabolism. In addition to lowering TG and increasing HDL-C, as mentioned above, pemafibrate improves fatty liver. These effects have been verified in basic studies, but few real-world data are available.

In daily clinical practice, clinicians still encounter many patients in whom triglyceride levels are difficult to correct, and many patients are left untreated because they are asymptomatic. Furthermore, hypertriglyceridemia is often caused by alcohol intake, but it is unclear whether patients with alcoholic hypertriglyceridemia have similar outcomes to those seen in trials of pemafibrate. Therefore, in this study, we collected information on the efficacy and safety of pemafibrate in patients with hyperlipidemia under routine medical care, with an additional focus on the subset of patients with alcoholic hypertriglyceridemia.

## 2 Methods

### 2.1 Patients

Participants were patients with high serum TG levels attending the outpatient clinic of Fukuoka University Chikushi Hospital or being treated by local physicians registered in the Chikushi Cardiovascular Clinical Research Network. Patients aged 20 years or older with hypertriglyceridemia, including alcoholic hypertriglyceridemia, who had started pemafibrate treatment within the past 12 weeks were included. Data obtained at the start of pemafibrate treatment were used as baseline data. Participants were enrolled in the study within 3 months after initiating treatment with pemafibrate, and written consent was obtained from all participants prior to their enrollment.

### 2.2 Exclusion criteria

The exclusion criteria were as follows: history of hypersensitivity to pemafibrate; high LDL-cholesterol only; severe liver damage (liver cirrhosis classified as Child-Pugh class B or C or biliary obstruction); renal dysfunction (serum creatinine value ≥ 2.5 mg/dL or creatinine clearance value < 40 mL/min); gallstones; pregnancy; and treatment with cyclosporine or rifampicin.

### 2.3 Discontinuation criteria

Patients discontinued their participation in the study if they withdrew their consent or were found to meet an exclusion criterion, or if a physician judged it inappropriate for them to continue the study.

### 2.4 Evaluation endpoints

The primary endpoints were changes in TG and HDL-C from baseline to 24 weeks. Secondary endpoints were changes in hepatic biomarkers and other blood test values (e.g., LDL-C, blood glucose, and renal function) and safety ＆ side effects during the study period. Patients were categorized into three drinking groups, i.e., daily drinking, occasional drinking, and non-drinking groups, on the basis of past alcohol use, and endpoints were compared between the three groups. Body weight monitoring was conducted before and after the 24th week of the test, excluding those with excessive weight changes.

### 2.5 Determination of LDL-cholesterol and remnant

Levels of LDL-C were measured directly. Remnant value was calculated by subtracting LDL-C from Non HDL-C (TC-HDL-C).

### 2.6 Determination of liver damage, alcohol amount and fibrosis-4 index (FIB-4)

We defined liver damage as either AST ≥31 IU/L or ALT ≥31 U/L, based on Japanese specific health checkup criteria. The amount of pure alcohol (g) was calculated as alcohol content (%)/100 × 0.8 (alcohol specific gravity) x 100. The average alcohol content of each alcoholic beverage was 5 % for beer, 15 % for sake, 43 % for whiskey/brandy, 25 % for shochu, and 12 % for wine. The FIB-4 index is a non-invasive and simple method for evaluating liver fibrosis in chronic liver disease [7]. FIB-4 index was calculated as (AST x age)/(platelet (10^9^/L) x √ALT).

### 2.7 Diabetes mellitus, hypertension and dyslipidemia

Diabetes, hypertension, and dyslipidemia were defined based on whether drugs were prescribed for each or according to Japanese guidelines [5,8,9].

### 2.8 Statistical analysis

Statistical analysis was performed at Fukuoka University with IBM SPSS Statistics version 23 (IBM Corp, Armonk, NY, US). Significant differences were tested by Student's *t*-test for items with normal variation and by the Mann-Whitney test for items without normality. Equality of variance was tested with the Levene test, and when equal variance was not assumed, Welch's test was performed. Correlation was tested with Spearman's rank correlation coefficient. Numerical results are expressed as mean ± standard deviation (SD), median with interquartile range (IQR), or frequency ratio. A p value of less than 0.05 was considered significant.

### 2.9 Ethical considerations

This study was performed after obtaining the approval of the Ethics Committee for Medicine of Fukuoka University (approval number: C20-07-009). The study protocol and patient informed consent forms were included in the ethics committee application documents, and written consent was obtained from each patient before enrollment. The study was conducted in accordance with the Declaration of Helsinki.

## 3 Results

A total of 203 patients were enrolled at 14 facilities from October 2020 to March 2022, and 29 patients were excluded (2 patients who experienced side effects, 1 patient who died from lung cancer, 15 patients who dropped out (9 not examined, 4 withdrawal from protocol, 2 withdrawal of the consent), 9 patients with non-fasting TG values, and 2 patients with large weight gain or loss of 15 kg or more), so data from 174 patients were analyzed. The dosage of pemafibrate was 0.2 mg per day in most cases (91.7 %), 0.1 mg per day in 12 cases, and 0.4 mg per day in 2 cases.

Patient background characteristics are shown in Table 1. The mean age was 65 years, and 64 % of patients were male. Common comorbidities were hypertension (approximately 77 %), diabetes (43 %), and high LDL-C (approximately 44 %). Statins were administered in 35 % of patients.

**Table 1:** Patient characteristics.

**Characteristics**	N (%)
N	174
Mean age (SD), y	65.5 ± 12.5
Male	112 (64.4)
Body mass index, kg/m2	26.1 ± 4.0
SBP, mmHg	131.4 ± 13.8
DBP, mmHg	76.4 ± 10.0
HR, bpm	74.9 ± 9.2

**Clinical presentation**	
Hypertension	134 (77.0)
Diabetes mellitus	75 (43.1)
Hyper LDL cholesterolemia	76 (43.7)
Hyperuricemia	51 (29.3)
Smoking	43 (24.7)
Drinking	96 (55.2)
Chronic kidney disease	21 (12.1)
Ischemic heart disease	8 (4.6)
Previous stroke	6 (3.4)

**Medication**	N (%)
Statin	61 (35.1)
EPA	6 (3.4)
Ezetimibe	15 (8.6)
ω-3 fatty acid	4 (2.3)

Data are numbers (%) or means ± standard deviation.

DBP, diastolic blood pressure; EPA, eicosapentaenoic acid; HR, heart rate; SBP, systolic blood pressure; N, number Medication demonstrate only drugs that target lipid.

**Fig. 1:**
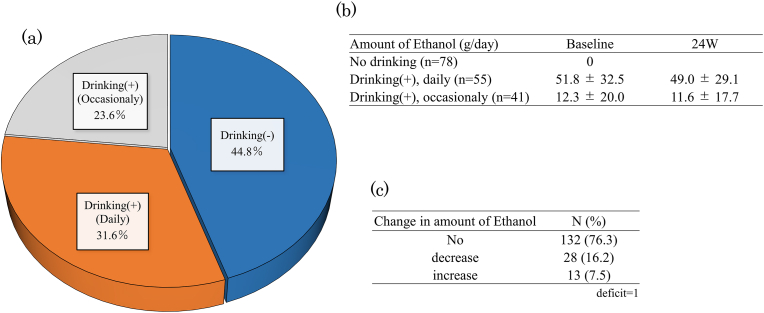
Drinking history at baseline (a), amount of ethanol at baseline and 24 weeks (b) and change in amount of ethanol at 24 weeks (c).

Alcohol drinking history and amounts are presented in Fig. 1. Alcohol drinking at baseline was seen in 55 % of patients, of whom 32 % consumed alcohol every day and 22 % consumed alcohol occasionally. In the daily drinking group, the mean daily amount of ethanol was 51.8 g.

At 24 weeks, 28 patients (16.2 %) showed a decrease in alcohol consumption, and 132 patients (76.3 %) showed no change in alcohol consumption.

Changes in evaluation parameters are shown in Table 2. Median TG was 284 mg/dL (IQR, 228–392 mg/dL) at baseline and decreased significantly to 141 mg/dL (IQR, 108–194 mg/dL) at 24 weeks (p < 0.01). Median HDL-C significantly increased from 46 mg/dL (IQR, 39–53 mg/dL) at baseline to 53 mg/dL (IQR, 45–60 mg/dL) at 24 weeks (p < 0.01), and mean LDL-C also significantly increased from 106.8 ± 31.3 mg/dL at baseline to 113.1 ± 27.2 mg/dL at 24 weeks (p = 0.01). Tests of hepatic biomarkers, such as aspartate transaminase (AST), alanine transaminase (ALT), alkaline phosphatase (ALP), and gamma-glutamyl transpeptidase (γGTP), significantly decreased after 24 weeks. No significant changes were observed in estimated glomerular filtration rate (eGFR) or creatine kinase (CK).

**Table 2:** Change in parameters at 24 weeks.

	Baseline	24 W	P value
Body weight, kg (n = 152)	69.7 ± 14.2	69.4 ± 14.4	0.67
HbA1c, % (n = 108)	6.6 ± 1.1	6.6 ± 1.1	0.40
Glucose, mg/dl (n = 165)	118.9 ± 39.8	121.7 ± 51.7	0.45
TC, mg/dl (n = 147)	207.5 ± 39.2	192.0 ± 29.1	<0.01
LDL-C, mg/dL (n = 161)	106.8 ± 31.3	113.1 ± 27.2	0.01
HDL-C, mg/dL, (IQR) (n = 163)	46 (39–53)	53 (45–60)	<0.01
Triglyceride, mg/dL, (IQR) (n = 173)	284 (228–392)	141 (108–194)	<0.01
Non-HDL-C, mg/dl (n = 143)	161.0 ± 40.3	138.9 ± 30.6	<0.01
Remnants, mg/dl (n = 134)	51.5 ± 26.2	24.4 ± 15.8	<0.01
AST, U/L, (IQR) (n = 172)	24 (21–34)	24 (20–30)	<0.01
ALT, U/L, (IQR) (n = 171)	26 (19.5–39)	20 (14–29)	<0.01
γ-GTP, U/L, (IQR) (n = 168)	42 (28–75)	26 (19–48)	<0.01
ALP, U/L, (IQR) (n = 130)	219 (171–255)	153 (126–183)	<0.01
Amy, U/L, (IQR) (n = 124)	70 (55–89)	66 (52–81)	<0.01
FIB-4 index	1.56 ± 0.83	1.56 ± 0.76	0.92
UA, mg/dl (n = 160)	5.9 ± 1.4	5.8 ± 1.3	0.26
eGFR, mL/min (n = 168)	68.6 ± 17.4	68.1 ± 17.1	0.41
Cr, mg/dl, (IQR) (n = 171)	0.84 (0.66–0.94)	0.83 (0.70–0.93)	0.77
WBC, × 103/μl (n = 169)	6.6 ± 1.6	6.6 ± 1.8	0.52
RBC, × 104/μl (n = 168)	473.6 ± 50.0	472.8 ± 54.4	0.74
Hb, g/dl (n = 169)	14.7 ± 1.4	14.5 ± 1.5	<0.01
Plt, × 104/μl (n = 165)	24.4 ± 6.1	25.9 ± 6.7	<0.01
CK, U/L, (IQR) (n = 151)	90 (64–124.5)	93 (65–120)	0.47
Na, mmol/L (n = 156)	139.9 ± 2.4	140.3 ± 2.0	0.95
K, mmol/L (n = 161)	4.3 ± 0.4	4.4 ± 0.5	0.01

Data are means ± standard deviation or medians (interquartile range [IQR]).

**Fig. 2:**
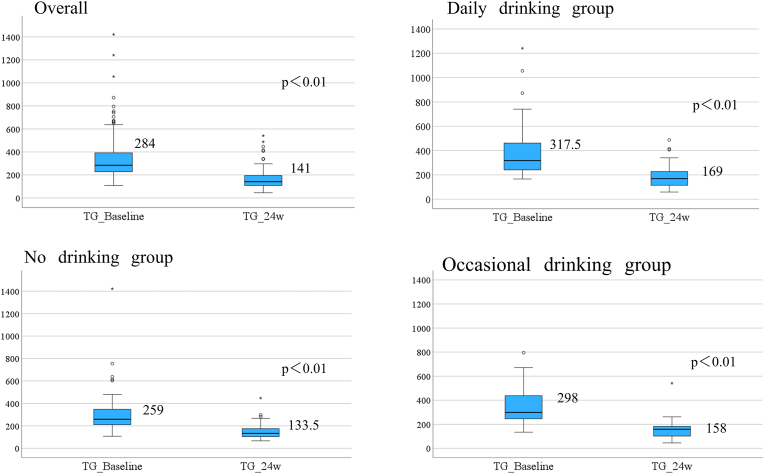
Change of triglyceride in relation to pattern of drinking history.

Changes in TG over time are shown for the overall population and by drinking group in Fig. 2. TG decreased significantly at 24 weeks in all three drinking groups. Baseline TG was highest in the daily drinking group, followed by the occasional drinking group.

Biochemical parameters are shown according to drinking group in Table 3. Baseline TG, body weight, γGTP, and uric acid were significantly higher in the daily drinking group, and the differences persisted even after 24 weeks. Differences in TG were also significant between the three groups at baseline and 24 weeks. Most hepatic biomarkers were lower at 24 weeks than at baseline in all three groups, although AST was lower after 24 weeks only in the daily drinking group.

**Table 3:** Change in parameters (a) and comparison (b) at 24 weeks according to drinking history.

	No drinking	Daily drinking	Occasional drinking	p value†
	group (n = 79)	group (n = 56)	group (n = 41)	
Body weight, kg				
Baseline	66.5 ± 12.7	72.6 ± 13.9	71.9 ± 16.6	0.02
24 W	66.5 ± 13.2	66.5 ± 13.2	71.5 ± 16.1	0.047
p value∗	0.96	0.84	0.13	
LDL-C, mg/dL				
Baseline	112.6 ± 30.1	99.2 ± 33.6	104.8 ± 24.7	0.05
24 W	109.7 ± 25.0	109.7 ± 25.0	118.7 ± 26.5	0.29
p value∗	0.37	<0.01	0.01	
HDL-C, mg/dL, (IQR)				
Baseline	45 (39–52)	48 (40–56)	43 (38–52)	0.20
24 W	51 (46–59)	51 (46–59)	53 (44–59)	0.36
p value∗	<0.01	<0.01	<0.01	
TG, mg/dL, (IQR)				
Baseline	259 (211–348)	318 (241–462)	298 (245–437)	0.01
24 W	134 (104–175)	169 (114–228)	158 (102–183)	0.04
p value∗	<0.01	<0.01	<0.01	
AST, U/L, (IQR)				
Baseline	23 (19–29)	29 (23–40)	22.5 (19–33)	<0.01
24 W	22 (20–27)	22 (20–27)	24 (18–29.5)	0.18
p value∗	0.30	<0.01	0.21	
ALT, U/L, (IQR)				
Baseline	25 (19–33)	28 (23–43)	24 (18–34)	0.07
24 W	20 (17–27)	20 (17–27)	17 (14–28)	0.60
p value∗	<0.01	<0.01	<0.01	
γ-GTP, U/L, (IQR)				
Baseline	32 (21–47)	71 (40–142)	43 (31–67)	<0.01
24 W	21 (15–29)	21 (15–29)	23.5 (19–48)	<0.01
p value∗	<0.01	<0.01	<0.01	
eGFR, mL/min				
Baseline	68.5 ± 16.8	68.4 ± 17.7	70.1 ± 18.3	0.78
24 W	67.5 ± 15.8	67.5 ± 15.8	68.7 ± 16.7	0.58
p value∗	0.28	0.53	0.21	
UA, mg/dl				
Baseline	5.5 ± 1.5	6.2 ± 1.3	6.0 ± 1.1	<0.01
24 W	5.3 ± 1.1	5.3 ± 1.1	6.2 ± 1.3	<0.01
p value∗	0.12	0.29	0.16	

Data are means ± standard deviation or medians (interquartile range [IQR]).

24 W, after 24 weeks of treatment; ALT, alanine transaminase; AST, aspartate transaminase; CK, creatine kinase; eGFR, estimated glomerular filtration rate; γ-GTP, gamma-glutamyl transpeptidase; HDL-C, high-density lipoprotein cholesterol; LDL-C, low-density lipoprotein cholesterol; UA, uric acid.

†p values were calculated for comparison of 3 categories.

∗p values were calculated for each parameter between baseline and after 24 weeks of treatment.

**Fig. 3:**
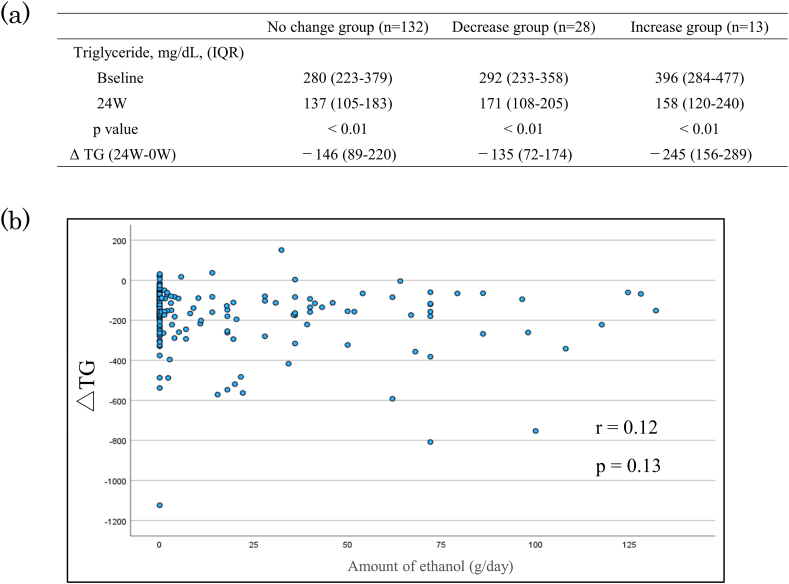
Changes of triglyceride according to changes in alcohol consumption at 24 weeks (a) and Relationship between ΔTG and Amount of ethanol (b).

The changes of Triglyceride according to changes in alcohol consumption at 24 weeks and the relationship between ΔTG (TG after 24 weeks - TG at baseline) and the amount of drinking are shown in Fig. 3. In the case where there was no change in alcohol consumption, TG at baseline was 280 (223–379) mg/dL, which decreased significantly to 137 (105–183) mg/dL at 24 weeks (p < 0.01). TG decreased significantly in both cases where alcohol consumption decreased and cases where alcohol consumption increased. ΔTG was not significantly correlated with alcohol consumption (p = 0.13, r = 0.12).

A stratified analysis of patients with and without statin use is shown in Table 4. LDL-C was significantly higher at week 24 than at baseline in the non-statin group but not in the concomitant statin group. No worsening of renal function or elevation of CK was observed regardless of whether statins were used or not.

**Table 4:** Change in parameters at 24 weeks with or without statin use.

	Statin (−) (n = 113)			Statin (+) (n = 61)		
	Baseline	24 W	P value	Baseline	24 W	P value
Alchol (g/day)	29.3 ± 40.7	26.4 ± 36.5	0.06	14.5 ± 31.6	13.4 ± 27.5	0.16
Alchol (%)	69 (58)			30 (49)		
Body weight, kg	70.3 ± 13.9	70.4 ± 14.1	0.71	68.6 ± 15.1	68.2 ± 15.0	0.01
LDL-C, mg/dL	109.2 ± 32.4	117.6 ± 29.5	<0.01	102.3 ± 29.0	104.8 ± 20.3	0.52
HDL-C, mg/dL, (IQR)	43.5 (38–52)	50 (44–58)	<0.01	46 (41–54)	55 (47–66)	<0.01
Triglyceride, mg/dL, (IQR)	299 (243–394)	159 (116.5–203)	<0.01	258 (211–391)	116 (89–174)	<0.01
AST, U/L, (IQR)	24 (20–35)	23 (19–31.5)	<0.01	24 (21–33)	25 (21–28)	0.13
ALT, U/L, (IQR)	26 (18.5–39)	20 (14–33)	<0.01	26 (20.5–38)	19 (14–26.5)	<0.01
γ-GTP, U/L, (IQR)	44 (28–86)	31 (20–60)	<0.01	39 (27–58)	23 (19–31)	<0.01
ALP, (IQR)	215 (164–242)	153 (132–180)	<0.01	219 (180–273)	144 (123–183)	<0.01
UA, mg/dl	5.9 ± 1.3	6.0 ± 1.3	0.92	5.7 ± 1.5	5.4 ± 1.2	0.09
eGFR,mL/min/1.73m2	69.7 ± 17.7	69.5 ± 17.5	0.75	66.5 ± 16.9	65.5 ± 16.2	0.28
CK, U/L, (IQR)	88 (62–120)	88 (64–114)	0.83	99 (71–130)	97 (68–149)	0.35

Data are means ± standard deviation or medians (interquartile range [IQR]).

ALP, alkaline phosphatase; ALT, alanine transaminase; AST, aspartate transaminase; CK, creatine kinase; eGFR, estimated glomerular filtration rate; γ-GTP, gamma-glutamyl transpeptidase; HDL-C, high-density lipoprotein cholesterol; LDL-C, low-density lipoprotein cholesterol; UA, uric acid.

Changes in hepatic biomarkers at 24 weeks in daily drinking group divided into those with and without liver damage, are shown in Table 5. Both AST and ALT were lower at 24 weeks than at baseline only in liver damage group.

**Table 5:** Change in hepatic biomarkers at 24 weeks with or without liver damage in daily drinking group.

	liver damage (+) (n = 31)			liver damage (−) (n = 24)		
	Baseline	24 W	P value	Baseline	24 W	P value
AST, U/L, (IQR)	37.0 (33.0–55.0)	30.0 (23.0–42.0)	<0.01	22.0 (21.0–27.0)	22.0 (18.0–25.5)	0.72
ALT, U/L, (IQR)	42.0 (32.0–68.0)	29.0 (19.0–43.0)	<0.01	22.5 (17.3–25.0)	16.0 (12.3–22.5)	0.046
γ-GTP, U/L, (IQR)	78.0 (51.0–150.0)	54.0 (34.0–66.0)	<0.01	59.0 (38.3–147.0)	41.0 (30.0–64.0)	<0.01
Plt, × 104/μl	21.3 ± 4.6	23.0 ± 5.3	<0.01	25.5 ± 7.0	26.4 ± 6.7	0.02

Data are means ± standard deviation or medians (interquartile range [IQR]).

ALT, alanine transaminase; AST, aspartate transaminase; γ-GTP, gamma-glutamyl transpeptidase; Plt, platelets.

Side effects were reported in 2 patients, including nausea and dizziness. These side effects did not lead to serious outcomes.

## 4 Discussion

This multicenter, open-label, prospective observational study showed that 24 weeks of pemafibrate administration significantly decreased TG and significantly increased HDL-C in patients with hypertriglyceridemia. In addition, hepatic parameters such as AST, ALT, γGTP, and ALP decreased. The patients in the present study were attending a hospital or local practitioner's clinic, so the study provides evidence for the effectiveness of taking pemafibrate to control lipids and decrease liver damage in routine clinical practice.

This study investigated the TG-lowering effect of pemafibrate according to alcohol use (no drinking, daily drinking, and occasional drinking). TG decreased significantly in all three alcohol use groups, and no significant difference was observed between groups in the extent of TG decrease. Furthermore, no correlation was observed between the extent of TG decrease and alcohol use at baseline. The most cases showed no change in alcohol consumption after 24 weeks, and even in these cases, TG decreased significantly. Although detail dietary habits and energy intake could not be evaluated, there were no significant changes in body weight or HbA1c at 24 weeks, indicating that there were no significant change in energy intake. In addition, when examining gender differences, baseline TG was significantly higher in men and baseline HDL-C was significantly higher in women, but there were no gender differences in the amount of TG change and HDL change at 24 weeks (data not shown). Pemafibrate itself was thought to be effective in cases of hyperlipidemia with high triglyceride levels, regardless of whether alcohol was consumed, energy consumption, blood sugar control, or gender.

The following processes have been reported as mechanisms by which alcohol increases TG: (1) Ethanol is broken down into acetaldehyde in the liver, which in turn produces acetic acid, and acetic acid supplies acetyl-coenzyme A and promotes fatty acid synthesis [10]; (2) production of radicals by acetaldehyde impairs mitochondrial function and inhibits AMP-activated protein kinase and promotes sterol regulatory element-binding transcription factor 1, increasing fatty acid synthesis [10,11]; and (3) ethanol is metabolized to acetaldehyde cytochrome P450 2E1, an enzyme that promotes oxidation, decreases PPARα activity, decreases fatty acid β-oxidation, and increases fatty acid production [10,12]. Pemafibrate, an SPPARMα, suppresses only process (3), but, our present date showed potent anti hypertriglyceridemia effect of pemafibrate in the alcohol induced TG increase suggests that PPARα activity is largely involved in the mechanism of alcohol induced TG increase. These consideration leads an ideas that pemafibrate is clinically useful. On the other hand, after 24 weeks, the TG value was the highest in the daily drinking group, and a TG value of less than 150 mg/dL was found in 62 % of the non-drinking group, 48 % of the occasional drinking group, and 38 % of the daily drinking group, i.e., the rate decreased as the drinking frequency increased. These results suggest that processes (1) and (2) for inhibiting TG elevation require abstinence from alcohol consumption. In other words, in patients with alcoholic hypertriglyceridemia, lipid management requires comprehensive management of drug treatment, restriction of alcohol intake through moderation or abstinence, and dietary therapy.

When participants were divided into three groups based on alcohol use, the baseline liver damage, hypertriglyceridemia, and hyperuricemia were already worse in the daily drinking group than in the non-drinking group. After 24 weeks, the differences in AST and ALT between these groups disappeared, and γGTP significantly decreased in the daily drinking group, indicating that pemafibrate decreased hepatic biomarkers even in patients drinking alcohol. Many studies have reported that pemafibrate suppresses TG synthesis in the liver and improves fatty liver [[13], [14], [15]], and it is clinically meaningful that pemafibrate decreases liver damage even in patients with alcoholic liver injury. When the fibrosis-4 (FIB-4) index, an index of liver fibrosis, was examined at baseline and 24 weeks, no significant changes in mean values were found. This lack of improvement in the FIB-4 index may be due to the fact that the study population had a low risk of liver fibrosis. Further research is required to determine whether pemafibrate improves the FIB-4 index and liver fibrosis in a population with a FIB-4 index score above the median value.

In this study, LDL-C was significantly elevated at 24 weeks after starting pemafibrate. Phase 2 [16] and large-scale clinical trials [17] of pemafibrate have shown that LDL-C increases after pemafibrate administration, and the present study showed similar results. LDL-C elevation was greater in patients with higher baseline TG (data not shown, p ≤ 0.01, r = 0.32). Pemafibrate inhibits TG synthesis in the liver and induces lipoprotein lipase (LPL) recruitment to promote TG catabolism, which may induce production of LDL-C from very low-density lipoprotein cholesterol, resulting in higher LDL-C levels and TG hypercatabolism. Pemafibrate has been reported to reduce levels of small dense LDL-C, which has a stronger pro-atherosclerotic effect than LDL-C [18], but normal LDL-C itself also causes atherosclerosis. One study also found that cardiovascular risk is higher in groups with higher levels of both small dense LDL-C and overall LDL-C [19], indicating that LDL-C control is also important.

In clinical practice, we experience some patients in whom serum TG is close to or above 400 mg/dL and serum LDL-C increases from less than 100 mg/dL to over 140 mg/dL after administration of TG-lowering drugs. We speculate that there is another reason for this effect in addition to TG hypercatabolism. In this study, the daily drinking group had significantly higher baseline TG and significantly lower baseline LDL-C than the non-drinking group. These results indicate that when TG levels are markedly high, a large amount of LPL is mobilized for TG metabolism and clearance of LDL-C may not be able to catch up, resulting in a lower LDL-C value that masks the original value. Considering that the higher the TG level is simultaneously the higher LDL-C levels may be, careful follow-up of LDL-C is required in patients with hypertriglyceridemia, regardless of the use of TG-lowering drugs. In other words, in these patients, it is important to manage not only TG itself, but also overall lipid metabolism, including LDL-C.

Boren et al. found in a large observational study that remnants are an atherosclerotic cardiovascular risk independent of LDL-C [20]. Remnants can be measured by lipoproteinophoresis or as remnant-like cholesterol, but these assays is difficult for local physicians to perform in practice. Instead, remnants can be calculated by subtracting LDL-C from non-HDL-C, and calculated remnants have been reported to be associated with the risk of death in patients with ischemic heart disease [21]. In this study, calculated remnants decreased significantly at 24 weeks, suggesting that pemafibrate may reduce remnants not only TG decrease and HDL-C increase and have a beneficial effect in lipid metabolism improvement.

In this study, a slight, non-significant increase in LDL-C was observed in the concomitant statin group. Conventionally, the combination of statins and fibrates is known to increase the incidence of rhabdomyolysis [22,23], but this study did not show any increase in CK. A phase III trial comparing pemafibrate with fenofibrate reported a smaller reduction in eGFR with pemafibrate [24], but the present study found no significant change in eGFR. Compared with conventional fibrates, pemafibrate is considered to have a lower risk of rhabdomyolysis and renal dysfunction, probably because it is a selective PPARα modulator of hepatic lipid metabolism and is excreted by the liver. As mentioned above, many patients with high TG levels have potentially low LDL-C levels, and the higher the TG level, the higher the possibility that statin therapy will be required. It is of great significance that we were able to demonstrate efficacy and safety in a real-world setting.

Triglyceride-lowering therapy with pemafibrate did not reduce cardiovascular events in the PROMINET trial [17]. On the other hand, a report [25] examining triglycerides and cardiovascular diseases risk in a large-scale cohort study combining the Atherosclerosis Risk in Communities Study and the Framingham Offspring Study suggested that the triglyceride cutoff value may be lower than 150 mg/dL. This report also showed that in patients with high-density lipoprotein cholesterol (HDL-C) < 40 mg/dL in the PROMINENT study, cardiovascular risk was reduced with a triglyceride of 100 mg/dL. In the PROMINENT study, the median triglyceride value after pemafibrate administration only decreased down to 189 mg/dL. These results suggest that if triglyceride is reduced to 150 mg/dL or even 100 mg/dL or less, the relationship with cardiovascular risk may differ from previous studies. In addition, the fact that non- HDL-C was hardly reduced in the PROMINENT study may also be a factor in the failure to reduce cardiovascular risk. It is known that basal triglyceride increases with alcohol intake, and the triglyceride level after the treatment with pemafibrate in this study may be insufficient in terms of reducing cardiovascular risk, but the important point of this study is that pemafibrate was effective in lowering triglyceride even in alcohol drinking cases.

## 5 Study limitation

First, although this was a single-arm, open-label study and a placebo effect cannot be ruled out, patient registration did not start until after treatment had started, which minimizes the placebo effect of drug administration itself. Second, some patients who definitely had an indication to be treated with statins did not receive statins. Third, it was impossible to identify the mechanism of LDL-C increase after administration of pemafibrate because it was a multicenter study by practitioners in clinical practice. To draw more accurate conclusions, further clinical trials that include additional analyses such as lipoprotein fractionation and LPL activity are required.

## 6 Conclusion

In this multicenter, prospective observational study in real-world clinical practice, pemafibrate led to improvements in TG, HDL-C and decreased hepatic biomarkers. Furthermore, pemafibrate was equally effective in improving TG regardless of alcohol use, including the amount of alcohol consumption. Although pemafibrate administration increased LDL-C, the increase was suppressed in patients receiving concomitant treatment with statins, and the study demonstrated the safety of combined therapy with pemafibrate and statins.

## CRediT authorship contribution statement

**Yosuke Takamiya:** Writing – original draft, Investigation, Formal analysis, Data curation. **Chiyori Imanaga:** Investigation, Data curation. **Amane Ike:** Supervision, Investigation. **Akira Kawamura:** Supervision, Investigation. **Hidenori Urata:** Writing – review & editing, Supervision, Methodology, Funding acquisition.

## Declaration of interest statement

The principal investigator of the study has received financial support from 10.13039/100015993Kowa Company, Limited (20-P06). None of the other authors have conflicts of interest to declare.
